# Early Cardiac Tamponade in a Patient with Postsurgical Hypothyroidism

**DOI:** 10.1155/2015/310350

**Published:** 2015-07-30

**Authors:** Archana Sinha, Sri Lakshmi Hyndavi Yeruva, Rajan Kumar, Bryan H. Curry

**Affiliations:** ^1^Division of Cardiology, Saint Luke's University Health Network, Bethlehem, PA 18015, USA; ^2^Division of Hematology and Oncology, Howard University Hospital, 2041 Georgia Avenue NW, Washington, DC 20060, USA; ^3^Division of Cardiology, Howard University Hospital, 2041 Georgia Avenue NW, Washington, DC 20060, USA

## Abstract

Pericardial effusion is a common cardiac manifestation of hypothyroidism, but effusion resulting in cardiac tamponade is extremely rare. We present a case of a 56-year-old African American woman with slurred speech and altered mental status that was initially suspected to have stroke. Her chest X-ray revealed cardiomegaly and subsequent echocardiogram showed a large pericardial effusion with echocardiographic evidence of cardiac tamponade. Clinically, patient did not have pulsus paradoxus or hypotension. Further questioning revealed a history of total surgical thyroidectomy and noncompliance with thyroid replacement therapy. Pericardiocentesis was performed promptly and thyroxine replacement therapy was started. Thereafter, her mental status improved significantly. The management of pericardial effusion associated with hypothyroidism varies depending on size of effusion and hemodynamic stability of the patient. The management strategy ranges from conservative management with close monitoring and thyroxine replacement to pericardiocentesis or creation of a pericardial window.

## 1. Introduction

Pericardial effusion may be caused by acute pericarditis, tumor, uremia, hypothyroidism, trauma, cardiac surgery, or other inflammatory conditions. Pericardial effusion is a known complication of hypothyroidism with the incidence ranging from 3–6% in mild cases of hypothyroidism to 30%−80% in severe hypothyroidism [1]. Cardiac tamponade secondary to hypothyroidism is rare as the fluid accumulates slowly, allowing for pericardial sac distension [2]. Even a small pericardial effusion can cause clinically significant tamponade, if it accumulates rapidly. It is important to suspect tamponade when patients have hemodynamic compromise regardless of the amount of pericardial effusion present. We report here a case of early cardiac tamponade, due to untreated postsurgical hypothyroidism.

## 2. Case Report

A 56-year-old African American woman with past medical history of hypothyroidism, hypertension, dyslipidemia, seizures, schizophrenia, and mood disorders was brought to emergency room by her estranged son for slurred speech and altered mental status. On further questioning, patient revealed a history of total surgical thyroidectomy done about ten years ago and admitted noncompliance with all her medications including thyroid replacement therapy for the last 4 years. She did not report any symptoms of constipation, cold intolerance, or weight gain. Her family history was unknown, social history was negative for any substance abuse, and she stayed alone.

**Figure 1:**
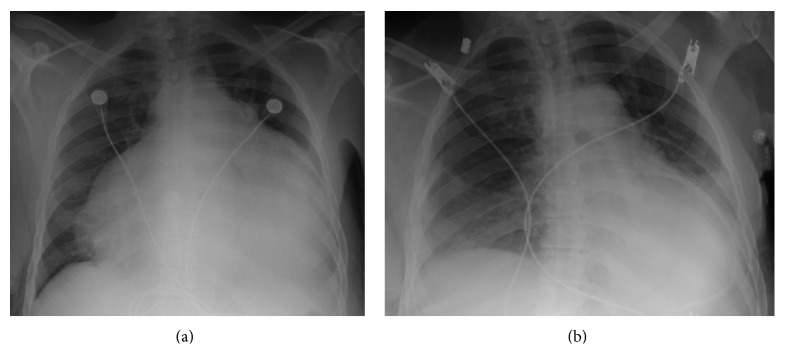
CXR. (a) Classical “water bottle” shaped cardiomegaly due to pericardial fluid. (b) Resolution of cardiomegaly after pericardial fluid drainage.

**Figure 2:**
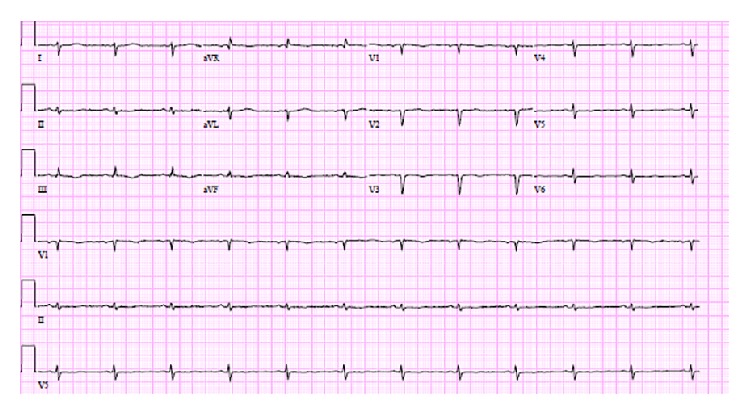
EKG. Low voltage QRS complexes.

**Figure 3:**
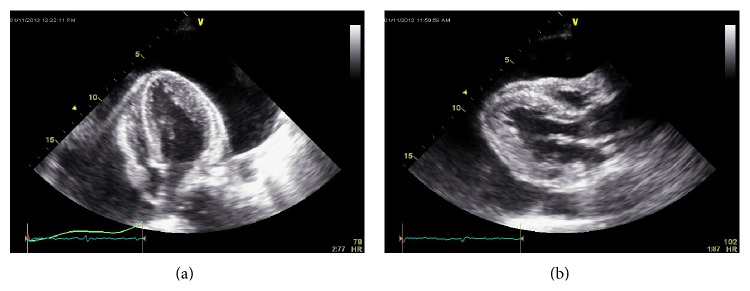
Echocardiogram. (a) Apical four-chamber view showing “swinging heart” and large pericardial effusion, (b) parasternal long axis view showing right ventricular diastolic collapse.

Her vitals on presentation were temperature of 98°F, blood pressure of 167/90 mmHg, pulse rate of 91 beats per min, and respiratory rate of 18 per min. Her physical exam showed dry skin, brittle hair, jugular venous distention, pulsus paradoxus < 10 mm of Hg, distant and muffled heart sounds, and delayed relaxation of ankle jerk. She also had slow slurred speech and slow mentation. In the emergency room, CT scan and MRI of head were negative for acute stroke. Her anteroposterior chest radiograph revealed massive cardiomegaly (Figure 1(a)). 12-lead Electrocardiography (EKG) revealed low voltage QRS complexes in all leads (Figure 2). A transthoracic echocardiogram was performed which showed ejection fraction of 55–60% and large (>3 cm) pericardial effusion with evidence of diastolic collapse of the right heart chambers (Figures 3(a) and 3(b)) and a significant respiratory variation of mitral and tricuspid inflow velocities. Cardiology consult was placed for further management.

Her blood chemistry panel was normal except for abnormal thyroid profile reflecting severe hypothyroidism with thyroid stimulating hormone of 89.33 mIU/L, total thyroxine (T4) of 5.15 nmol/L, and total triiodothyronine (T3) of 0.46 nmol/L. Her erythrocyte sedimentation rate (ESR) was 104 mm/Hg. She was started on levothyroxine 75 mcg, with plan to up-titrate it to optimal dose depending on clinical tolerability.

**Figure 4:**
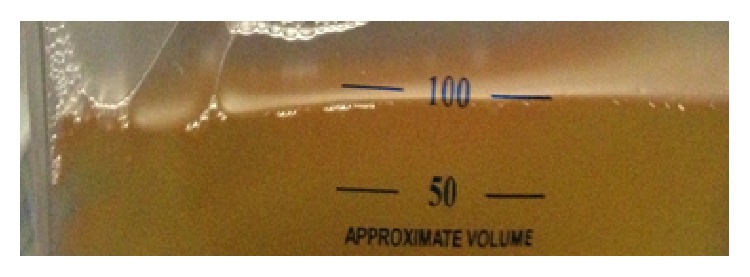
“Gold paint effusion” of hypothyroidism.

Transthoracic echocardiogram guided pericardiocentesis via the subxiphoid approach was performed. Samples of the hazy, yellow pericardial fluid were sent for biochemistry, cytology, microscopy, and sensitivity testing. Therapeutic pericardiocentesis was performed and pericardial drain was left in place. Patient's hemodynamic status was continuously monitored, during and after the pericardiocentesis. Lim et al. have shown association of fatal postprocedural “pericardial decompression syndrome” with pericardiocentesis [3]. Three liters of hazy, yellowish/“gold paint” pericardial fluid was drained (Figure 4).

The patient's vital signs remained stable and a repeat chest X ray (Figure 1(b)) and echocardiogram revealed that the pericardial effusion had decreased considerably in size. Pericardial fluid analysis was performed (Table 1). Pericardial fluid bacterial and fungal cultures were negative. Her creatinine phosphokinase was 527 U/L and troponin I was negative. Her ANA titre was positive at 1 : 1280 with centromere pattern. ANA titer can be false positive in 5% of tested women and elderly and can also be high in patients with thyroid disease. Rheumatoid factor, ds DNA, SM, RNP, and nucleosomal chromatin antibody were all negative. We ruled out all other possible etiologies of pericardial effusion. Hypothyroidism was the only abnormality that could explain the large pericardial effusion and subsequent early tamponade in our patient. She was started on thyroxine supplementation, which improved her mental status and speech considerably. She was discharged with outpatient follow-up with endocrinology and cardiology.

## 3. Discussion

The British physician C. Parry in 1785 was the first to report the deleterious effect of excess thyroid hormone on heart, who described heart enlargement as a consequence of hyperthyroidism [4]. H. Zondeck showed that hypothyroidism could cause cardiac enlargement (pericardial effusion) along with decrease pulsations and low electrocardiographic voltage, which was subsequently called “myxedema heart.”

Cardiovascular abnormalities are common in those with abnormal thyroid functions and changes include abnormal cardiac contractility, heart rate, and peripheral vascular resistance [5]. Pericardial effusion can be a common finding in hypothyroidism but an effusion large enough to cause cardiac tamponade is rare with only few reports in English literature [6–11].

The rare occurrence of cardiac tamponade can be attributed to the pericardial distensibility and the slow accumulation of fluid, allowing significant fluid accumulation without hemodynamic compromise. Even though heart failure is unusual in hypothyroidism, it should be differentiated from tamponade because of similar presentations [12]. The volume of pericardial effusion is directly related to the duration and severity of the hypothyroidism but there has been no correlation with thyroid stimulating hormone levels and the existence or severity of the effusions. The factors responsible for development of pericardial effusion in hypothyroidism include increased capillary permeability with efflux of proteins rich fluid and glycosaminoglycans into the pericardial sac decreased lymphatic drainage and high retention of salt and water [13].

The diagnosis of hypothyroidism as the cause of cardiac tamponade in emergent situations at times can be difficult because of its slow onset, nonspecific signs, and symptoms [14].

Pericardial effusions associated with hypothyroidism have an insidious onset and initially present without significant hemodynamic changes; if left untreated, development of effusion into cardiac tamponade can be explained in a sequential manner. Initially there is moderate to large pericardial effusions without tamponade, followed by echocardiographic tamponade without paradoxical pulse and finally when pericardial pressure remains continuously above the intracavitary pressure, overt hemodynamic collapse develops [15]. Our patient had echocardiographic evidence of tamponade but lacked clinical signs of tamponade. This scenario might be present very early in the tamponade physiology. Even though the pericardial pressure transiently exceeds the intracavity pressure, cardiac output may still be maintained owing to the very slow rate of pericardial fluid accumulation.

The usual presenting clinical triad of cardiac tamponade which includes hypotension, distant heart sounds, and jugular vein engorgement, popularly referred to as Beck's triad, may be present or absent. On the contrary, our patient was noted to be hypertensive, which could be explained by profound vasoconstriction, as a result of sympathetic overactivity. Previous studies have shown that subacute to chronic tamponade may present with hypertension in approximately one-third of patients, especially in those with preexisting hypertension and renal failure [16]. The absence of sinus tachycardia in patients diagnosed with cardiac tamponade should arouse strong suspicion of hypothyroidism [17]. Bradycardia can also be seen in cardiac tamponade as a consequence of hypothyroidism. It has been suggested that it can be due to decreased sympathetic activity, but indirect measurements of sympathetic activity have showed it to be elevated. There is some evidence of blunted sympathetic excitatory and tachycardic response to hypotension, and depressed arterial baroreflex, with elevated dependence on the resting sympathetic tone that could explain the presence of bradycardia [18]. Wang et al. studied hypothyroid pericardial effusion and recommended that if heart rate is approximately 80 beats per minute in hypothyroid patients with echocardiographic signs of tamponade, clinical tamponade is evident and possibly warrants emergent drainage [15].

The EKG findings include decreased voltage with electrical alternans, T wave flattening which has been mentioned to be associated with myxedema heart disease [15]. QT prolongation has also been observed in patients with hypothyroidism and tamponade likely secondary to the hypothyroidism. The presence of sinus bradycardia, low QRS voltage, diffuse flat T waves, and long QT has positive predictive value for hypothyroidism [19]. Echocardiogram (M or 2D mode) is the preferred investigation for diagnosis of cardiac tamponade. The findings suggestive of cardiac tamponade on echocardiography include pericardial effusion, diastolic right atrial and right ventricular collapse, inferior vena cava dilation, and loss of respiratory variations and respiratory increase of interventricular dependence. Left ventricle “pseudohypertrophy” can also be present due to increased diastolic wall thickness. This can be seen as an overall consequence of hypothyroidism. Hypothyroidism gives rise to arterial wall stiffness and induces hypertension, which if not treated can produce LV concentric hypertrophy [20, 21].

The management of cardiac tamponade is guided by the hemodynamic status of the patient. Early tamponade with only mild hemodynamic compromise may be treated conservatively. Thyroxine replacement is the mainstay of treatment along with fluid restriction and close observation. In patients with severe hemodynamic compromise needle pericardiocentesis or surgical drainage with creation of a pericardial window is employed. In such individuals, quick removal of pericardial fluid is imperative because there is direct relationship between volume of pericardial fluid and pericardial pressure. Removal of as little as 50 mL of pericardial fluid can produce appreciable hemodynamic and symptomatic improvement. Conscious effort should be made to avoid measures that can decrease venous filling pressures and can negatively affect cardiac output [22]. Echocardiogram guided pericardiocentesis is well tolerated by patients and can be quickly performed even in unstable patients. The success rate is close to 97% and the preferred route of approach is the subxiphoid region [23]. The recurrence of effusion after pericardiocentesis has been observed in the past and this should be anticipated while draining effusion caused by hypothyroidism [24].

## 4. Conclusion

Our patient did not present with typical symptoms of clinical tamponade and severe hypothyroidism posing clinical dilemma. A high index of suspicion must be maintained for timely diagnosis of cardiac tamponade due to severe hypothyroidism, followed by prompt intervention. All the patients diagnosed with cardiac tamponade without sinus tachycardia or with bradycardia should be worked up for hypothyroidism. The management of cardiac tamponade caused by hypothyroidism is different from other causes of tamponade, cases with mild hemodynamic compromise can be managed conservatively with thyroxine replacement, and those with severe hemodynamic compromise need needle pericardiocentesis or surgical drainage.

## Figures and Tables

**Table 1:** Pericardial fluid analysis.

Pericardial fluid	Our patient	Normal
Appearance	Hazy	Clear
Color	Golden yellow	Pale yellow
Glucose	80 mg/dL	106–159 mg/dL
LDH	150 mg/dL	276–517 mg/dL
Total protein	5.5 g/dL	2.8–4.8 g/dL
WBC	51	0–5
Polymorphs	73	None
RBC	101	0–20
Mesothelial cells	Few	None
Adenosine deaminase	4.7	<9.2
Specific gravity	1.035	—
Cholesterol	68 mg/dL	<55 mg/dL
